# Association of Body Mass Index and Diet with Symptomatic Gall Stone Disease: A Case-Control Study

**DOI:** 10.7759/cureus.7188

**Published:** 2020-03-05

**Authors:** Qamar Kiani, Fareeha Farooqui, Muhammad Sohaib Khan, Aoun Z Khan, Muhammad Nauman Tariq, Aisha Akhtar

**Affiliations:** 1 General and Colorectal Surgery, Shifa Tameer-E-Millat University, Islamabad, PAK; 2 Surgery, Shifa Tameer-E-Millat University, Islamabad, PAK; 3 General Surgery, Shifa International Hospital, Islamabad, PAK; 4 Surgery, Rawalpindi Medical University, Islambad, PAK; 5 Internal Medicine, Holy Family Hospital, Rawalpindi, PAK; 6 Surgery, Texas Tech University Health Sciences Center, Lubbock, USA

**Keywords:** cholelithiasis, gall stone disease (gsd), diet, bmi

## Abstract

Background: Gall stone disease (GSD) is one of the commonest gastrointestinal disorders. Causative factors can be non-modifiable as genetics and modifiable like dietary habits. High-fat diet and high BMI are well known contributing factors world-over. Local and regional data is deficient about these factors. Moreover, Indo-Pakistani dietary patterns are very different from the western population. This study was conducted to see the association of high BMI and diet with GSD in our population.

Materials and Methods: This case-control study was conducted in Shifa International Hospital, Islamabad, from 2012 to 2017. We included all female patients above 25 years of age with symptomatic gall stones on ultrasonography as cases and all females above 25 years of age with no gall stones on ultrasonography (done for any reason) as controls. The patients with gall stones having some other clear predisposing cause for gallstone formation, e.g., stoma, sudden weight loss, etc. were excluded. BMI, demographic details, dietary habits, and clinical profile of cases and controls were recorded. Data were analyzed by SPSS v 21. p-values were calculated.

Results: A total of 396 patients were included in this study, with 103 cases and 293 controls. The mean BMI of GSD cases was 27.576±5.753, while controls had a mean BMI of 25.638±7.008 (p=0.08). About 26.4% of cases had an average fat consumption greater than 100g/day as compared to 11.9% controls (p=0.035). The average red meat intake per week was 222g among cases while 210g among controls (p=0.001). The average intake of fried food per week was 270g among controls and 250g among cases (p=0.012). The average intake of bakery items per week was 240g among cases and 210g among controls (p=0.038).

Conclusion: Gall stone disease is significantly related to high BMI and high dietary fat and meat intake in our population.

## Introduction

Gall stone disease (GSD) is one of the commonest gastrointestinal pathologies presented in a health care setting [1]. The incidence of GSD is increasing with the prevalent sedentary lifestyle [2]. In a study conducted in Pakistan, the surgical incidence for cholelithiasis was found to be 9.03%, with females being 3.3 times more prone to develop gallstones than males [3]. Cholelithiasis is associated with serious and fatal complications like acute cholecystitis, choledocholithiasis, choledochal fistulae, pancreatitis, and gall bladder cancer [4]. It also costs the health care system a lot, in the form of cholecystectomies. Hence, knowledge of modifiable etiological factors is vital to reduce the burden on the health care system and avoid associated life-threatening complications.

GSD is multifactorial in origin, including genetic and dietary factors [5]. Many risk factors are associated, such as weight, age, dyslipidemia, chronic liver disease, gender, and parity [6]. Among these, a high-fat diet is a known modifiable risk factor, as is obesity [6]. Complicated GSD has been reported to be strongly associated with metabolic syndrome and diabetes mellitus as well [7,8]. There are a lot of studies from the western countries showing association of gall stones with cooking oils, refined sugars, meat, and margarine [9,10]. Also, studies show the association of GSD with a high-fat diet [9]. Obesity, rapid weight changes, and physical activity are also associated with the formation of gall stones [10]. Most of these studies are from western countries regarding dietary intake and GSD, and there is no local study from this region showing association of gall stone disease with BMI and diet. Moreover, dietary patterns and eating habits in this region are different from the western population. The changing epidemiology necessitates the identification of target populations for future therapies. An improved etiological and pathophysiological understanding of gallstone disease may lead to novel, non-surgical options for prevention and therapy. There is a lack of local published data in developing countries on this issue, studies in these countries could substantiate the existing evidence and provide valuable new information. Based on this, we can decrease the disease burden by increasing awareness about the modifiable risk factors, especially diet and the body mass index. So, we conducted this study to identify the association of GSD with certain dietary intake and BMI in our population.

## Materials and methods

A case-control study was conducted in the Department of Surgery, Shifa International Hospital, Islamabad, from the year 2012 to 2017. Cases were all adult female patients of symptomatic gall stones disease diagnosed by clinical symptoms and confirmed on ultrasonography. A convenient consecutive sampling technique was used. The controls were all adult females admitted at Shifa International Hospital for complaints other than cholelithiasis and with normal abdominal ultrasound done for any other reason. The study was performed after approval was granted by the ethics committee of Shifa International Hospital, and informed consent was obtained from all participants (participation=96%). Each participant selected for the study was personally interviewed in the inpatient department. Patients were interviewed regarding their demographic details, dietary habits of the last five years, and clinical profile on admission. Data were recorded in a predesigned proforma, and variables recorded included pregnancy, diabetes mellitus, oral contraceptive use, hyperlipidemia, menopause, family history, alcohol, cirrhosis, recent weight loss, chronic kidney disease, and Hepatitis B and C status. In terms of dietary habits, both groups were compared for the consumption of red meat, chicken, fish, eggs, ghee, fried food, cakes, biscuits, cream, and dry fruits. BMI of both controls and cases were calculated and recorded from height and weight charts. Data were entered in SPSS v 21. Descriptive statistics were calculated, and the chi-squared test was applied. 

## Results

**Figure 1:**
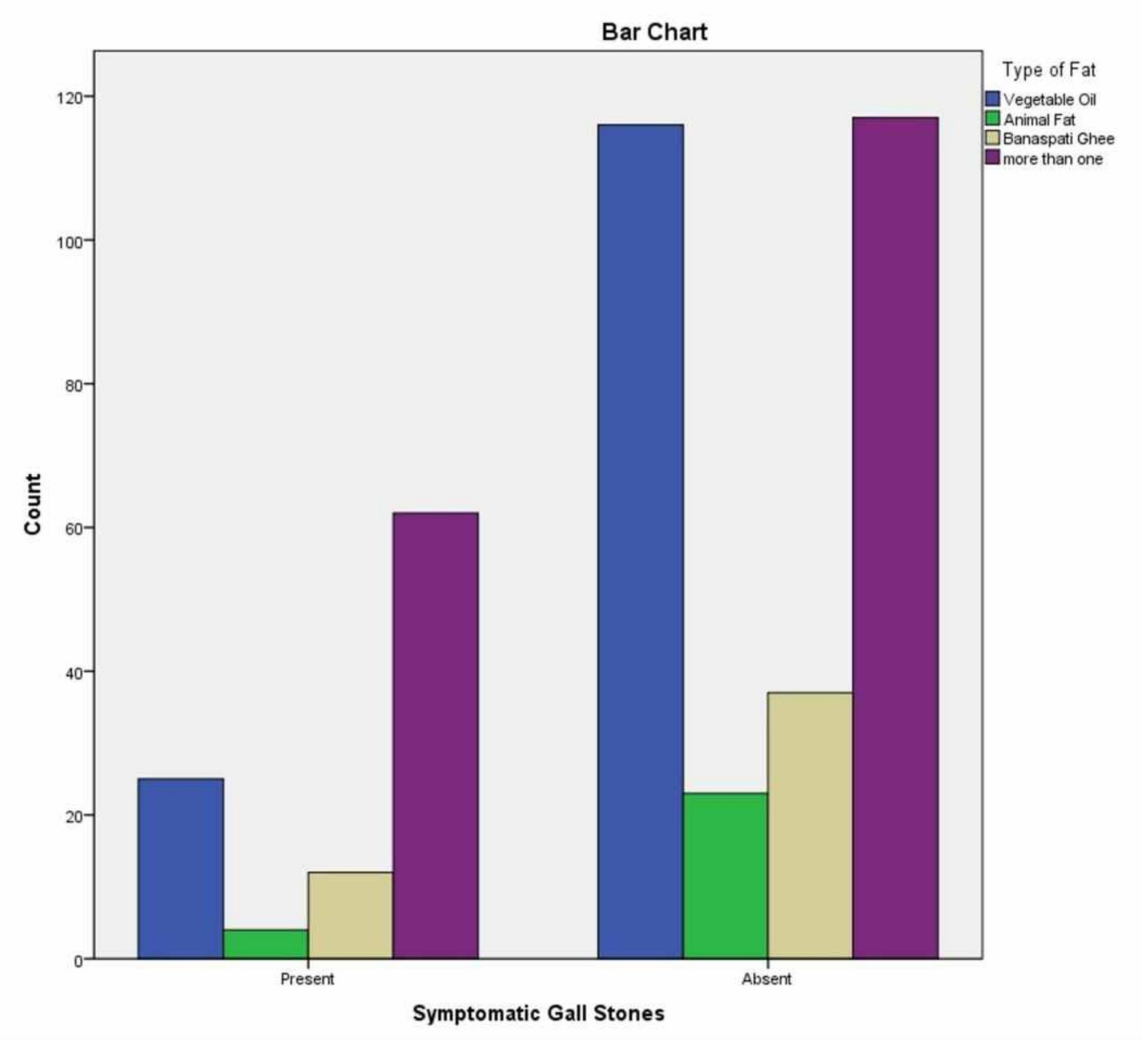
Association of gall stone disease with the type of fat intake

**Figure 2:**
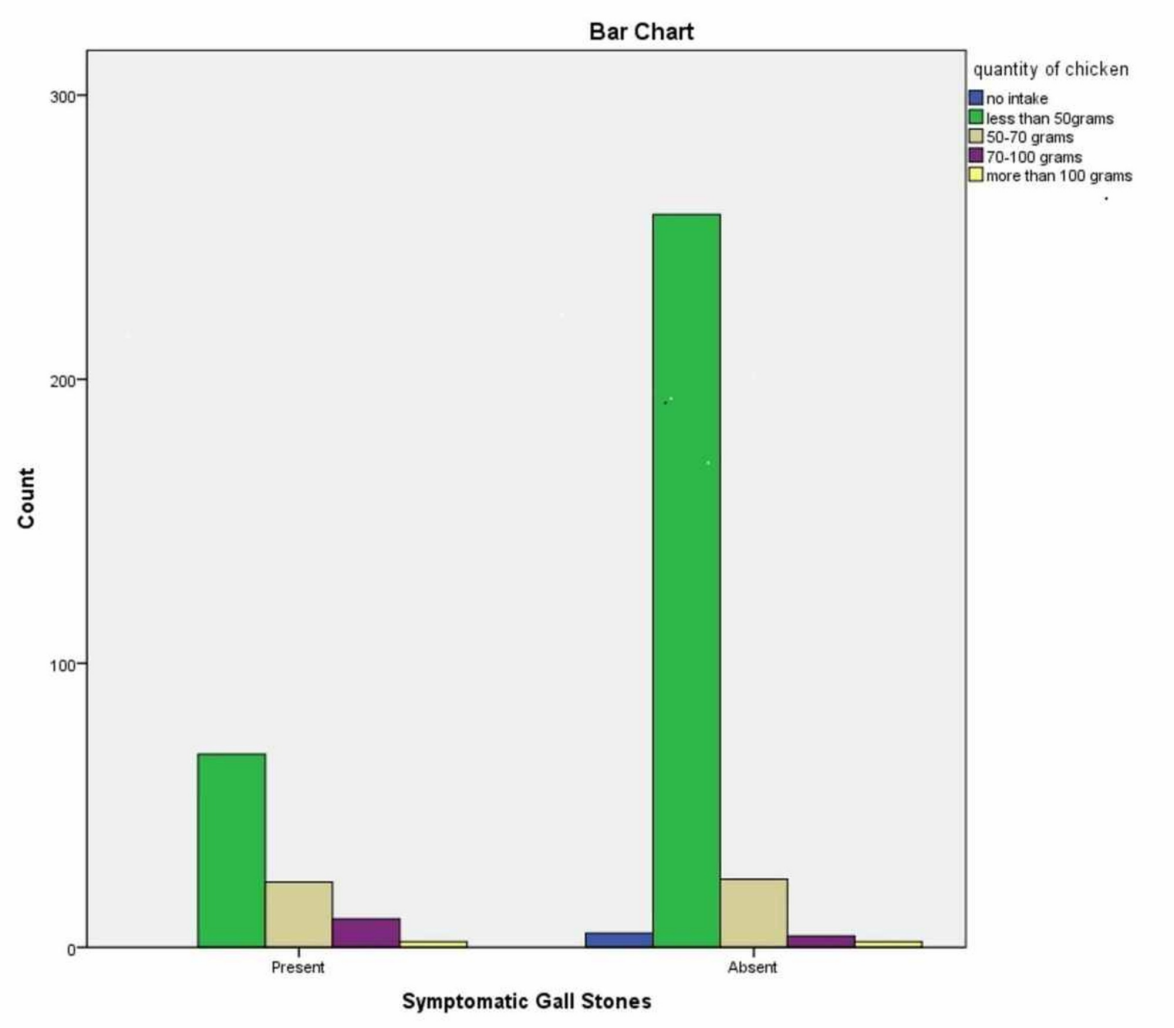
Association of fat intake with the quantity of chicken intake

A total of 396 patients were included in this study, out of which 103 were cases, and 293 were controls. All patients were females above the age of 25 years; the maximum age was 84 years. Mean BMI of GSD cases was 27.576±5.753 kg/m^2, ^while controls had a mean BMI of 25.638±7.008 kg/m^2^ (p=0.08). Mean parity was 3.01±2.513 of both cases and controls; 25.3% of the controls were nulliparous as compared to 14.6% of cases (p=0.016). The average fat consumption higher than 100g/day was reported by 26.4% of cases as compared to 11.9% controls (p=0.035). The average red meat intake per week was 222 g among cases while 210 g among controls (p=0.001). The average chicken intake per week was 343 among cases and 255g among controls (p=0.019). The average consumption of fried food per week was 270g among controls and 250g among cases (p=0.012). The average intake of bakery items per week was 240g among cases and 210g among controls (p=0.038). The average consumption of dry fruits per week was 102g among cases and 90g among controls (p=0.00). Rests of the dietary factors studied were not significantly different between the cases and the controls. The association of GSD with the type of fat intake and quantity of chicken has been shown in Figures 1, 2, respectively.

Table 1 shows the different p-values of various dietary and non-dietary factors among cases and controls.

**Table 1:** Percentages and p-values of various dietary and non-dietary factors among cases and controls

Variables		Percentage of Cases	Percentage of Controls	p-value
Pregnancy		8.7%	9.2%	0.885
Diabetes Mellitus		14.5%	17.4%	0.505
Oral Contraceptives		6.8%	7.2%	0.899
Smoking		6.8%	8.5%	0.578
Hepatitis B or C		18.4%	11.3%	0.063
Known Hyperlipidemia		22.3%	15.7%	0.127
Menopause		45.6%	23.5%	0.000
Family History of Gall stones		43.7%	29.3%	0.008
Insulin Therapy		5.8%	3%	0.208
Cirrhosis		6.8%	4.4%	0.347
Chronic Kidney Disease		5.8%	6.1%	0.907
Recent acute weight loss		7.8%	8.5%	0.809
Ileostomy or Colostomy		0%	0.3%	0.553
Multiparity		37%	23%	0.000
Duration of insulin use		5.8%	3.7%	0.419
Average fat Intake >100g/day		26.4%	11.9%	0.035
Average red meat intake >50gm/week		74.8%	87%	0.000
Quantity of red meat >50gm/week		33%	20.8%	0.000
Quantity of Chicken >50gm/week		34%	10.2%	0.000
Quantity of Fish >50gm/week		12.6%	4%	0.007
Quantity of Egg >2/day		5.8%	3.4%	0.271
Quantity of Butter/ Ghee>100gm/week		3.8%	1.7%	0.442
Quantity of Fried Food>50gm/week		76.7%	22.86%	.001
Quantity of Cake/Biscuits>1 biscuit/day		80.6%	79.5%	.046
Quantity of Cream>50gm		5.8%	1.7%	0.025
Quantity of Dry Fruits≥50gm/week		85.4%	76.1%	0.000
Type of fat	Vegetable oil	24.2%	39.6%	0.003
	Animal fat	3.8%	7.8%	
	Banaspati ghee	11.6%	12.6%	
	More than one type of cooking oil and ghee	60.2%	40%	

## Discussion

Cholecystectomies lead to a major burden on the health care system. GSD also leads to many complications, which can be life-threatening as well, like acute pancreatitis and carcinoma [7,11]. An important physical factor affecting the development of gallstones studied in this article was BMI; the mean BMI of GSD cases was 27.576 kg/m^2^ while controls had a mean BMI of 25.638 kg/m^2^. Stender et al. indicated that the risk of symptomatic gallstone disease increased by 7% for every 1-kg/m^2^ increase in measured BMI [1]. Ben-Menachem also concluded in his study that obesity has a positive relationship with the development of gall stone [11]. Obesity is accompanied by increased synthesis and excretion of cholesterol into bile, wherein the amount of cholesterol produced is directly proportional to being overweight [12]. In our study, 23% of the controls were multiparous as compared to 37% cases (p-value=0.000), which is similar to the study by Naeem et al. conducted in Karachi in which high parity was found among the cholelithiasis patients [13]. Figueiredo et al. observed that the highest risk of GSD was present among women who reported having a child before age 20 [14]. We did not record the age at first parity in our group of patients. In our study, among the cases, 45.6% had menopause as compared to 23.5% of controls (p=0.000). Similarly, a study in Taiwan also concluded that menopause is a risk factor for cholelithiasis in females [15]. Another study suggested that postmenopausal estrogen therapy was associated with an increased risk of gallstone disease in current and former estrogen users [16].

In our study, we did not find a significant association of known hyperlipidemias with GSD (p=0.127). However, Lee et al. concluded that increased serum leptin concentrations and hyperlipidemia (hypercholesterolemia or hypertriglyceridemia) are associated with canine cholelithiasis, and that homeostatic imbalance of these parameters might affect the pathogenesis of gallstones [17]. We were unable to see a significant association of hyperlipidemias in our group of patients; the reason for this could be that we did not check the lipid profile of all cases and controls rather, we only asked the history of known hyperlipidemias. In our study, 18.4% of the cases were hepatitis B/C positive as compared to 11.3% of controls (p=0.063), while 6.8% cases had a history of liver cirrhosis as compared to only 4.4% of controls (p=0.347). Hung et al. found that hepatitis B and C infection and cirrhosis as risk factors for cholelithiasis in both males and females [15]. Acalovschi showed that gallstones often occur in patients with liver cirrhosis, and their prevalence increases with age and with the duration of disease [18]. Similarly, another study revealed a significant association between HBV infection and the risk of gallstones, and this was not observed in this study [19]. We found that a family history of gallstones was significantly related to GSD (p=0.008). Similarly, other studies like Nakeeb et al. suggest that the genetic factors account for at least 30% of the etiology of symptomatic GD [20].

Different dietary factors were compared between the cases and the controls; 26.4% cases had an average fat intake higher than 100gm/day as compared to 11.9% controls ( p=0.035), which are comparable to literature. Total fat, saturated, and monounsaturated fatty acids high intakes are associated with increased GD risk [21]. Higher intake of saturated fat or trans-fatty acids was associated with an increased incidence of gallstones [22]. We found that the average red meat intake of more than 50gm per week was significantly associated with GSD (p=0.000). The quantity of chicken intake more than 50gm per week was also significantly related to gall stone disease in our group of patients (p=0.000), as mentioned in Table 1. The average intake of fried food more than 50gm per week was also significantly related (p=0.001). Park et al. concluded that the consumption of red meat and animal lipid was positively associated with the risk of cholesterol gallstone [23]. Catala et al. showed that dietary proteins affect biliary cholesterol concentration and precipitation and gallstone formation [24]. In our study, the intake of bakery items was not found significantly related to the development of GSD, 80.6% cases vs. 79.5% controls (p-value =0.46). While another study showed that cholesterol level rises in the blood with the consumption of saturated fats leading to a higher risk of cholesterol stone formation in patients consuming butter, the reason for this controversy could be the change in diet after diagnosis of cholelithiasis [25]​​​​​​​.

Our study had a few limitations. We interviewed the patients after the development of the gall stones; they might have modified the diet after the development of gall stones. Secondly, it is difficult to recall the last few year’s food habits exactly. We recommend that further prospective dietary intake studies should be conducted in healthy patients who have other risk factors of GSD, like hereditary. We can then avoid the serious, life-threatening complications of GSD like acute pancreatitis and gall stone cancers by modifying these factors.

## Conclusions

Dietary factors and BMI are strongly associated with gall stone disease, along with positive family history and menopause. Future prospective studies should be conducted to see the association of dietary factors with the development of GSD.
